# Thalli Growth, Propagule Survival, and Integrated Physiological Response to Nitrogen Stress of *Ramalina calicaris* var. *japonica* in Shennongjia Mountain (China)

**DOI:** 10.3389/fpls.2018.00568

**Published:** 2018-05-08

**Authors:** Chuan-Hua Wang, Ming Wang, Rao-Zhen Jia, Hua Guo

**Affiliations:** ^1^Hubei International Scientific and Technological Cooperation Center of Ecological Protection and Management in the Three Gorges Area, China Three Gorges University, Yichang, China; ^2^Engineering Research Center of Eco-environment in the Three Gorges Reservoir Region, Ministry of Education, China Three Gorges University, Yichang, China

**Keywords:** nitrogen deposition, nitrogen metabolism, epiphytic lichens, growth and survival, Shennongjia nature reserve

## Abstract

In this study, effects of nitrogen (N) availability on growth, survival of *Ramalina calicaris* var. *japonica*, and whether it respond nitrogen stress in an integrated physiological way was evaluated. Thalli growth and propagule survival, thalli N and phosphorus (P) content, and activity of phosphomonoesterase (PME) of *R. calicaris* var. *japonica* were determined in a field experiment. Its differentiate adsorption in ammonia and nitrate, the activity of glutamine synthetase (GSA) and nitrate reductase (NRA) also were investigated in a series of indoor experiments. The results showed that N deposition significantly decreased the growth and survival of this lichen, and the N sensitivity threshold was suggested at 6.0 kg N⋅ha^-1^⋅y^-1^. When the N deposition increased from 8.59 kg N⋅ha^-1^⋅y^-1^ to 14.24, 20.49, 32.99 and 57.99 kg N⋅ha^-1^⋅y^-1^, the growth rates of lichen thalli decreased by 26.47, 39.01, 52.18 and 60.3%, respectively; Whereas the survival rate of the lichen propagules decreased from 92.8% of control (0.0 kg N⋅ha^-1^⋅y^-1^) to 10.7% of 50.0 kg N⋅ha^-1^⋅y^-1^, when they were treated with 0.00, 6.25, 12.5, 25.0, and 50.0 kg N⋅ha^-1^⋅y^-1^ deposition. Compared with an adequate adsorption of ammonium N, no nitrate adsorption occurred when thalli was submerged in solution lower than 0.4 mM. Our results also suggested that thalli total nitrogen, N:P ratio increased with N availability, and the activity of PME was significantly correlated with thalli total nitrogen. These all indicated that phosphorus limitation occurred when *R. calicaris* var. *japonica* treated with higher nitrogen deposition. Compared with slightly effects of NRA, GSA of *R. calicaris* var. *japonica* responded nitrogen availability significantly; In addition, GSA and NRA negatively correlated with thalli growth rate and propagule survival significantly. These results indicated that nitrogen stress do decrease growth and survival of *R. calicaris* var. *japonica*, and lichen would be impacted by excess nitrogen in a integrated, not a fragmentary way, including nitrogen uptake, assimilation, even nutrient balance of nitrogen and phosphorous.

## Introduction

Lichens mainly acquire mineral nutrients, including nitrogen (N) and phosphorus (P), directly from the atmosphere and rainfall via thalli surface, so they have evolved into a class of organisms sensitive to environmental pollution (Pearson and Skye, 1965; Galloway et al., 2004). Since 1840’s, the active N deposition in the global terrestrial ecosystem has increased 3.6 times (Galloway et al., 2004), which has led to a significant reduction in the plant diversity, especially in lichens (Geiser and Neitlich, 2007; Clark and Tilman, 2008).

The Shennongjia region is one of the main habitats for golden snub-nosed monkeys (*Rhinopithecus roxellana*), an endangered species on the Chinese red list of endangered animal species (Wang, 1998). Some epiphytic lichens are important fallback food for golden snub-nosed monkeys, accounting for approximately 40–60%, and even as high as 90%, of the diets of this monkey (Li, 2001, 2006; Guo et al., 2007). Among the lichens, *Usnea luridorufa, Usnea longissima, Ramalina sinensis* have been identified as food for golden snub-nosed monkeys (Li, 2006; Guo et al., 2007). A randomized sampling demonstrated absence of significant eutrophication in the area, but provided, along one of the main roads crossing the area, records of species like *Xanthomendoza ulophyllodes* and *Phaeophyscia ciliata,* that are characteristic for dust deposition and nutrient rich bark (Munzi et al., 2015). In this region, the wet N deposition in 2015 was 7.99 kg⋅ha^-1^⋅y^-1^ (Yang et al., 2018), which greatly exceeded the nitrogen deposition threshold of N-sensitive lichens of 3.2 kg⋅ha^-1^⋅y^-1^ (Fenn et al., 2008).

*Ramalina calicaris* var. *japonica*, a green algae lichen (Wu, 1987), is a dominant species among the epiphytic lichens in Shennongjia Mountain, China (Yang et al., 2017). It also exhibits quite strong N sensitivity in a previous research (Wang et al., 2015). Therefore, N deposition might have a significant impact on growth and survival of *R. calicaris* var. *japonica* in the Shennongjia region. Up to now, besides some preliminary researches on lichen taxonomy in this region, in-depth studies on the growth and survival response of these lichens to increasing N deposition is still scarce.

Lichenologist and ecologist have made many but fragmentary progresses to understand the nature of lichen sensitivity/tolerance to excess nitrogen. It is reported that less nitrogen tolerant lichens can uptake more ammonium nitrogen than nitrophytic lichens (Miller and Brown, 1999; Gaio-Oliveira et al., 2004; Hauck, 2010), and toxicity of ammonium on membrane integrity, photosynthetic performance, chlorophyll chemistry of sensitive lichens also were recognized as factors leading to species specific nitrogen tolerance (Munzi et al., 2009a,b, 2012, 2014). In addition, P-limitation, indicated by an activity elevation of phosphomonoesterase (PME), occurs when nitrogen addition is performed in some sensitive lichens (Hogan et al., 2010a,b). Hauck (2010) further suggests that carbon skeletons supplied by phytobiont are relevant to lichen’s ammonium tolerance, as the ability to provide sufficient carbon skeleton is correlated with ammonium tolerance in vascular plants (Schortemeyer et al., 1997). In summary, present theories to understand lichen nitrogen sensitivity/tolerance were diverse and fragmented, as the experiments are performed on different lichen species. Above all, species specific ability to assimilate ammonium and nitrate nitrogen were ignored at all in lichen nitrogen sensitivity studies, although high activities of glutamine synthetase (GS) and nitrate reductase (NR) have been proved crucial in producing tolerance to high ammonium concentrations in vascular plants (Cruz et al., 2006; Stevens et al., 2011).

We hypothesized that nitrogen stress would decrease growth and survival of physiologically sensitive lichens, and lichens would respond the stress in an integrated way from nitrogen uptake to metabolism. *R. calicaris* var. *Japonica*, an indigenous and N-sensitive dominant epiphytic lichen of Shennongjia Mountain, provided a perfect material to clarify whether N deposition will impact lichens growth and survival, and whether lichen will respond nitrogen stress in a integrated way, including a higher ammonium adsorption than nitrate, increased N, P content of thalli and their unbalance between them, elevation of the activity of PME, GS, and NR. Answering to these questions will contribute to understand the mechanisms underlying N sensitivity of lichens, also will be helpful in the management of winter food of golden snub-nosed monkeys in the Shennongjia Region.

## Materials and Methods

### Study Sites

The sampling site, mainly covered with mixed broadleaf-conifer forest (Tie et al., 2010), locates in a valley of Hongping Town of Shennongjia Region (31°34′N, 110°23′E, 1700 m altitude). The annual average temperature of this region is about 12°C, with an annual precipitation ranging from 1000 to 1800 mm (Tie et al., 2010). Background wet N load of the site is about 7.99 Kg ha^-1^ y^-1^, and nitrate and ammonia accounted for 40.75 and 44.08% of the total nitrogen of rainfall, respectively (Yang, et al., 2018).

### Impacts of Nitrogen Addition on Thalli Growth, N, P Content, and PME

#### Nitrogen Treatment

Following methods of Johansson et al. (2011), five 1.2 m × 2.0 m experimental plots were established in a relatively open canopy gap in the study site. Each plot was equipped with a 1.5 m height wooden rack, on which an irrigation tube with 18 small sprinklers was fixed at 1.2 m height. A timer (ZYT02-M, Shanghai Zhuoyi Electronic Co., Ltd., China) precise to second level was used to control the spray schedule and duration of N addition.

In March 2015, healthy *R. calicaris* var. *Japonica* thalli with similar size were collected and numbered, and initial air dry weight (m_1_R) of each individual was measured using the reference sample method of McCune et al. (1996). Among the samples, ten individuals were randomly selected as *reference samples*, and stored at -18°C to determine the final dry mass of *test samples*.

To test the effects of N addition on lichen growth, N and P content, five deposition levels were designed to simulate future nitrogen loads, from no pollution to values largely above the suggested critical loads for forest ecosystems (Air Pollution Information System [APIS], 2013). The five plots were randomly assigned to the five N treatments. In each plot, 20 thalli were glued with clear silicone sealant to nylon monofilament loops, then were fixed on the wooden racks and irrigated for 18 s per day, with either natural rain water (nitrogen concentration is ∼0.097 mM, which was equal to a load of 0.5–0.6 Kg N ha^-1^ y^-1^), or NH_4_NO_3_ solutions 0.41, 0.81, 1.63, and 3.2 mM (which were prepared with deionized water). These solutions and spraying time can simulate 0.6, 6.25, 12.5, 25.0, and 50.0 Kg N ha^-1^ y^-1^ N addition precisely. Treatments lasted from April 1 to October 31, 2015. During the irrigation time, the precipitation amount was 1519 mm, and about 226 L water was used on each spraying unit, which was equal to a precipitation of 94.5 mm, and only accounted for 6.45% of total precipitation per year.

#### Determination Final Biomass of *Test Samples*

The *Reference Method* of McCune et al. (1996) was used to determine the final biomass of test samples. Upon finishing the N treatment, the test samples, together with reference samples, were air-dried at room temperature for 48 h, then their air dry weight (m_2_) were measured with a balance (CPJ303, OHAUS Corporation, United States). For subsequent analysis, test samples were transported to China Three Gorges University and stored in a -18°C refrigerator. The *reference samples* were oven-dried at 65°C, then dry weight of each individual was determined, after which initial and final water content of *reference samples* were calculated, then were used to calibrate the initial and final dry mass of the *test sample*s (m_1_’ and m_2_’). Growth rate of each individual was calculated as: (m_2_’-m_1_’)/m_1_’ × 100%. In total, 20 replicates (thalli) were performed for each nitrogen treatment.

#### Determination of Ammonium N, Nitrate N, and Phosphorous Contents of Lichen Thalli

After finishing test 1.2.2, three lichen individuals were collected from one treatment, oven-dried and milled into powder, respectively, then three replicates were conducted for each nitrogen treatment. For each sample, about 500 mg of lichen material was digested with H_2_O_2_-H_2_SO_4_. Then phosphorus concentration (PC) was determined by phosphorus molybdenum heteropoly acid (PMA)-Malachite green spectrophotometry methods at 650 nm (Yang et al., 2018), nitrate, ammonium, and total N concentration (NC) in the digestion was determined with a San^+++^ auto analyzer (Skalar, Breda, Netherlands).

#### Assay of PME Activity

Phosphomonoesterase activity of lichen thalli was determined as the method of Turner et al. (2001). Briefly, in each nitrogen treatment, three lichen individuals were randomly selected from test 1.2.2, a piece of thallus of 2 cm length was cut from each thalli, then was added to 2.9 mL of 0.02 M citric acid–trisodium citrate solution. Then, 0.1 ml *para*-nitrophenylphosphate (*p*NPP) was added to the solution to reach a final concentration of 3 mM. Samples were subsequently incubated for 20 min at 15°C in a shaking water-bath in the dark. The reaction was terminated by transferring 2.5 ml of the essay solution into 0.25 ml terminator solution (1.1 M NaOH, 27.5 mM EDTA and 0.55 M K_2_HPO_4_). The absorbance was measured at a wavelength of 405 nm using a spectrophotometer (UV-5500PC, Metash instruments Co., Ltd., China). Thalli were then oven dried for 24 h at 65°C and weighed. Enzyme activity was expressed as μmol substrate hydrolysed g^-1^ dry mass h^-1^ using *p*-nitrophenol to calibrate the assay. Three replicates were performed for each nitrogen treatment.

### Adsorption of Ammonium and Nitrate

A single factor experiment was designed in this study. The factors were KNO_3_ and (NH_4_)_2_SO_4_. Considered the total N concentration of precipitation in Shennongjia, 0.02, 0.05, 0.1, 0.2, 0.4, 0.8, 1.6, and 2.0 mM of KNO_3_ and (NH_4_)_2_SO_4_ solutions were prepared with CaSO_4_ solution (0.2 mM) to study adsorption of ammonium and nitrate of *R. calicaris* var. *japonica*.

Forty lichens were activated in a climatic chamber for 7 days under conditions of 15°C, 40∼60 μmol⋅m^-2^⋅s^-1^ and RH 95% (Johansson et al., 2012), then they were randomly divided into eight groups. One branch of each thalli (approximately 50 mg) was rinsed with deionized water, and immersed into a calcium sulfate solution (CaSO_4_, 0.2 mM) for 24 h to remove the residual N in free space. Upon draining the surface water, the sample was immersed and shaked in a tube with 10 mL of N solution for 30 min (Dahlman et al., 2004). In total, each nitrogen solution had five replicates. Ammonium and nitrate concentration of the post-treated solution were determined as the methods of Yang et al. (2016). Finally, sample branches were oven-dried at 65°C, and their dry masses were measured.

The N adsorption rate (*A*) was calculated as following: *A* = (*N*_t_-*N*_0_)/*m*, where *N_t_* is the ammonium or nitrate concentration of the post-N-treated solution, and *N*_0_ is the ammonium or nitrate concentration of the pre-treated solution, *m* was the mass of lichen branche.

### Effects of N Deposition on Propagule Survival

Following methods of Armstrong (1990) and Sillett et al. (2000), *Salix fargesii* chips (5 cm × 8 cm) with bark were prepared to host lichen fragments. To that, epiphytes were removed carefully, then the chips were oven dried 24 h at 120°C to kill the organisms on the surface. Samples of *R. calicaris* var. *japonica* were manually crushed, and passed through a 2 mm and 5 mm mesh screen to ensure 2∼5 mm semi-natural size. Sixty lichen fragments were evenly sowed on each chips, then 20 chips were randomly divided into five groups (corresponding to five nitrogen treatments), each with four replicates. Each group was placed in a transparent plastics box and sprayed with artificial rainwater. The rainwater was prepared as in Johansson et al. (2010), which obtained 0.0, 0.05, 0.1, 0.2, and 0.4 mM NH_4_NO_3_. Considered the actual nitrogen concentration of our study site is about 0.097 mM (Yang et al., 2018), then above solutions could cover the actual and future nitrogen concentrations. Nitrogen deposition (0.0, 6.0, 12.5, 25.0, and 50.0 Kg N ha^-1^ y^-1^) was simulated through control the volume of the sprayed solution. The chips were sprayed every 2 days. All the boxes were placed in a climatic-chamber at 15 ± 2°C, RH 60 ± 5% and photoperiod of 12 h at 60∼80 μmol m^-2^ s^-1^ photons PAR. After 6 months, the survival of the lichen fragments was surveyed with microscope, fragments with normal color similar to reference lichens were considered healthy.

### Effects of Nitrogen Deposition on GSA and NRA

#### Assay of GSA

Six groups of lichens were incubated 2 months under a condition, same to the steps described in Section “Adsorption of Ammonium and Nitrate.” We sprayed lichens every 3 days with one of 0.1, 0.2, 0.4, 0.8, 1.6, and 2.0 mM NH_4_NO_3_ solution. Glutamine synthetase activity (GSA) was measured as Zhao et al. (2002). In detail, 50 mg air dried lichen was manually grounded with 2 ml 5 mM phosphoric acid buffer (containing 0.5 mM EDTA, 50 mM K_2_SO_4_, pH 7.2), then the buffer was centrifuged 20 min (18000 rpm, 4°C). Subsequently, 1.2 ml crude enzyme liquid, 0.6 ml imidazole-HCl buffer (0.25 M), 0.4 ml glutamic sodium (0.30 M),0.4 ml ATP-Na (30 mM), 0.2 ml MgSO_4_ (0.5 M) were added into a tube. The tube was incubated in a 25°C water bath for 5 min, then 0.2 ml hydroxylamine (0.5 M) was added in. Fifteen minutes later, 0.8 ml ferric trichloride solution was added to terminate the reaction. We centrifuged the reaction solution 15 min (4500 rpm, 4°C), and absorption value (A) of the supernate was measured at 540 nm with spectrophotometer (UV-5500PC, Metash instruments Co., Ltd., China). Relative GSA was presented as A⋅h^-1^⋅g^-1^⋅DW, in which A was the absorption value of supernate. Three replicates were performed for each nitrogen treatment.

#### Assay of NRA

Nitrate reductase activity (NRA) was determined as the methods of Zhao et al. (2002). About 50 mg lichen apex was submerged in to a tube containing 10 ml solution of 0.10 mol⋅L^-1^ KNO_3_. Then the tube was vacuumized 30 min, subsequently N_2_ was supplied into the tubes. Thereafter, tube was incubated in a dark, 25°C chamber for 30 min. Then enzyme reaction was terminated by taking the lichen apex out of the tube. Absorption of reaction solution was measured with spectrophotometer at 540 nm (UV-5500PC, Metash instruments Co., Ltd., China), as well dry mass of the lichen apex was measured. NRA was expressed as mg NO_2_^-^⋅h^-1^⋅g^-1^⋅DW. Each nitrogen treatment was replicated three times.

### Statistics

The effects of nitrogen availability on thalli growth, propagule survival, thalli nitrogen and phosphorous content, activity of GS, NR, and PME were analyzed with one-way ANOVA using the SPSS (IBM Corp. Released 2016. IBM SPSS Statistics Version 24.0). Differences were considered significant when *P* < 0.05. Comparisons of means were performed using a Tukey’s (HSD) test. Lichen adsorption response to nitrogen availability was plotted with Origin 7.5, and then its *K*_m_ and *V*_max_ were fitted with Michaelis–Menten model (V = $V_{\max}^{*}$C/(K_m_ + C)) (Turner et al., 2001). Finally, correlations of above data, in addition with nitrogen availability, were analyzed with SPSS (IBM Corp. Released 2016. IBM SPSS Statistics Version 24.0).

## Results

### Effects of N Deposition on Thalli Growth and Propagule Survival

**FIGURE 1:**
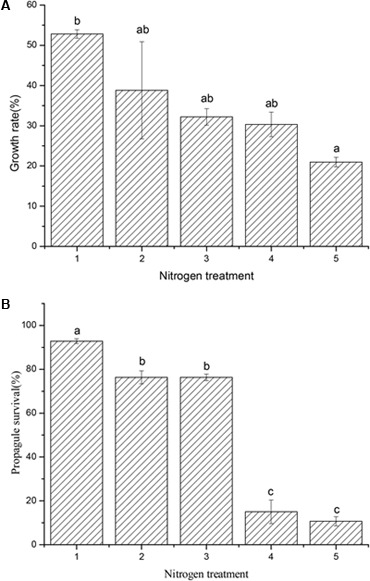
Effects of nitrogen addition on growth (**A**, *n* = 20) and propagule survival (**B**, *n* = 4) of *Ramalina calicaris* var. *japonica*. **(A)** code of horizontal axis represented 8.59, 14.24, 20.49, 32.99, and 57.99 kg N ha^-1^. y^-1^, respectively; **(B)** code of horizontal axis represented 0.0, 6.25, 12.5, 25.0, and 50.0 kg N ha^-1^ y^-1^, respectively. Vertical bars indicate standard deviation. Different lowercases indicate significant differences using the Tukey test (significance level at *p* < 0.05).

The results showed that the growth of *R. calicaris* var. *japonica* decreased when N deposition increased. Under N deposition of 8.59∼57.99 kg N ha^-1^.y^-1^, the growth rate of *R. calicaris* var. *japonica* declined from 52.8% (control, 8.59 kg N ha^-1^.y^-1^) to 20.97% (57.99 kg N ha^-1^.y^-1^). Accordingly, the extent of decrease under these N depositions relative to the control were 26.47, 39.01, 52.18, and 60.3%, respectively (see **Figure 1A**). At the same time, the survival of lichen propagules decreased from 92.8% (0.0 kg N ha^-1^.y^-1^) to 10.7% (50 kg N ha^-1^.y^-1^), indicating that a huge decrease (88.5%) of propagule survival occurred actually (**Figure 1B**). In general, except the treatments between 6.0 and 12.5 kg N ha^-1^.y^-1^, significant effects of other nitrogen depositions on survival were observed, then 6.0 kg N ha^-1^.y^-1^ could be deduced as the N-sensitive threshold for this lichen.

### Effects of N Deposition on N, P Content of Lichen Thalli

The results showed that N addition had significant effects on ammonium N (NH_4_^+^-N), total N (TN), total P (TP) and the N:P ratio (*p* < 0.05), but no effects on the nitrate content (*p* = 0.34) (see **Table 1**). In particular, except treatment of 14.24 kg N^-1^ ha^-1^ y^-1^, the NH_4_^+^-N, nitrate, TN, and TP of lichen all increased along the 8.59∼57.99 kg N^-1^ ha^-1^ y^-1^ treatments. Meanwhile, the N:P ratio always were greater than 10 (varied between 12.27 and 26.96 under the five nitrogen treatments), indicating that the lichen was in a constant state of N-induced P deficiency (see **Table 1**).

**Table 1:** ANOVA analysis of nitrogen addition on thalli nitrogen and phosphorous accumulation of *Ramalina calicaris* var. *japonica* (Mean ± SD, *n* = 3).

Nitrogen treatment	Ammonium (mg g^-1^)	Nitrate (mg g^-1^)	Total nitrate (mg g^-1^)	Total phosphorous (mg g^-1^)	N:P Ratio
(kg N^-1^ ha^-1^ y^-1^)				
0.60	3.99 ± 0.31a	8.68 ± 0.37a	12.67 ± 0.38b	0.96 ± 0.04b	12.27 ± 0.68a
6.25	3.14 ± 0.37a	8.39 ± 0.45a	10.23 ± 0.22a	0.62 ± 0.03a	16.41 ± 0.68c
12.5	3.46 ± 0.20a	8.70 ± 0.73a	12.12 ± 0.26b	0.88 ± 0.02b	13.78 ± 0.58b
25.0	5.54 ± 0.25b	9.08 ± 1.37a	17.52 ± 0.33c	1.13 ± 0.02c	15.56 ± 0.12c
50.0	5.19 ± 0.15b	9.67 ± 1.55a	16.71 ± 0.15c	0.62 ± 0.02a	26.97 ± 0.86d
*p*	0.00	0.341ns	0.00	0.00	0.00

*For one variable, data with different lower case letter was significant at p = 0.05 level, “ns” means there are no significant difference among treatments*.

### Response of PME to Nitrogen Deposition

**FIGURE 2:**
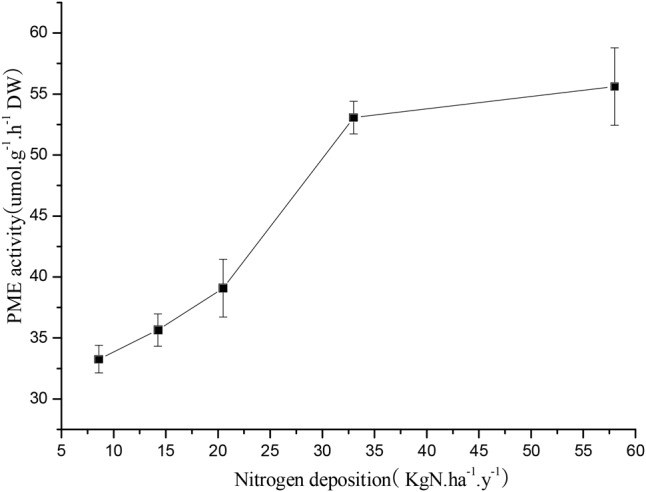
Response of phosphomonoesterase (PME) of *R. calicaris* var. *japonica* to nitrogen deposition (Mean ± SD, *n* = 3).

An elevation of PME of *R. calicaris* var. *japonica* thalli occurred when they were treated with increasing nitrogen availability. In detail, when lichens were treated with the 14.24 kg N a^-1^ ha y^-1^, the PME was 35.65 μmol h^-1^ g^-1^ (**Figure 2**). Then, PME increased to 55.62 μmol h^-1^ g^-1^ when treated with the 57.99 Kg N ha^-1^ y^-1^ N solution.

### Adsorption of Ammonium N and Nitrate N

**FIGURE 3:**
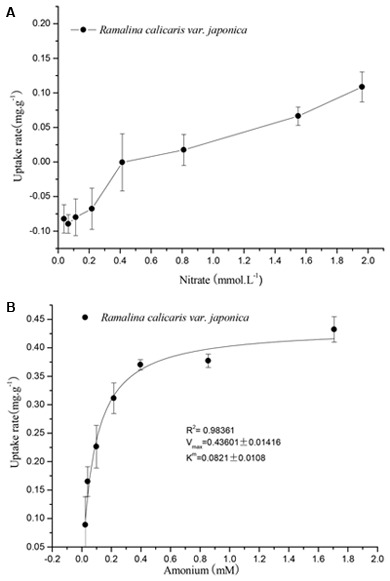
Response of nitrate **(A)** and ammonium **(B)** uptake of *R. calicaris* var. *japonica* to nitrogen availability (Mean ± SD, *n* = 5).

The results showed that the capacity of *R. calicaris* var. *japonica* to adsorpt nitrate N was much lower than that of ammonium N (see **Figure 3**). The NO_3_^-^-N adsorption rate was negative at the concentration of 0.1–0.4 mM, suggesting that there are no nitrate N adsorption occurred under the present nitrogen environment of precipitation in Shennongjia Mountain. The adsorption of nitrate N increased as its concentration increased, but it cannot be fitted by the model of Michaelis–Menten equation. When the concentration of the solution was greater than 0.4 mM, the NO_3_^-^-N adsorption rate changed from negative to positive, finally reached a maximum value of approximately 0.1 mg.g^-1^. In contrast, the adsorption of ammonium N by *R. calicaris* var. *japonica* fitted the Michaelis–Menten equation well (*R*^2^ = 0.9836, *p* < 0.01). The turning point occurred at an ammonium concentration of 0.2 mM, where the maximum adsorption rate (*V*_max_) was 0.436 mg.g^-1^. So its *V_max_* was four times of that of nitrate N. In addition, the Michaelis–Menten constant (*K*_m_) of ammonium was 0.082 mM, which indicated that *R. calicaris* var. *japonica* could utilize ammonium N at a very low concentration.

### Response of GSA and NRA to Nitrogen Deposition

**FIGURE 4:**
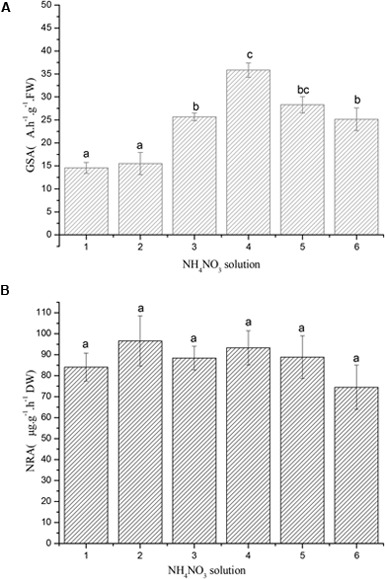
**(A)** Response of glutamine synthetase activity (GSA) of *R. calicaris* var. *japonica* to nitrogen addition (Mean ± SD, *n* = 3). Code of horizontal axis represented 0.1, 0.2, 0.4, 0.8, 1.6, and 2.0 mM NH_4_NO_3_ solution, respectively. Vertical bars indicate standard deviation. Different lowercases indicate significant differences using the Tukey test (significance level at *p* < 0.05). **(B)** Response of nitrate reductase activity (NRA) of *R. calicaris* var. *japonica* to nitrogen addition (Mean ± SD, *n* = 3). Code of horizontal axis represented 0.1, 0.2, 0.4, 0.8, 1.6, and 2.0 mM NH_4_NO_3_ solution, respectively. Vertical bars indicate standard deviation. Different lowercases indicate significant differences using the Tukey test (significance level at *p* < 0.05).

Our results suggested that GSA of *R. calicaris var. Japonica* was significantly impacted by nitrogen deposition. In general, response of GSA to increasing nitrogen dose showed an initial increase, then decrease curve (See **Figure 4A**), it demonstrated the highest activity (35.85 A h^-1^ g^-1^ DW) at 0.8 mM, which was 2.46 times of the controls. GSA decreased when nitrogen solution were over 0.8 mM, suggesting that lichen was damaged under such nitrogen deposition strength. In contrast, our results proved that NRA was not impacted by increased nitrogen deposition (**Figure 4B**).

### Correlations

**Table 2** showed the significant correlation coefficients (*Pearson*) among lichen variables. Lichen growth had strong correlations with the N provided, GSA, and NRA. Survival of propagules had a strong correlation with the N provided, the total nitrogen of thalli, the activity of PME and GSA. Activity of PME strongly correlated with the ammonium content, the total nitrogen content, GSA and NRA of thalli. In addition, GSA correlated with the nitrogen provided and NRA. Except above correlations, NRA, N:P ratio, nitrate content of thalli also strongly correlated with N provided.

**Table 2:** Correlations (Pearson) of *R calicaris* var. *japonica.*

	N:P Ratio	Nitrate	Total phosphorous	Total nitrogen	Growth rate	PME	Survival	GSA	NRA
N Addition	0.913P^∗^	0.952P^∗^	Ns	ns	–0.887P^∗^	ns	–0.903P^∗^	0.916P^∗^	0.897P^∗^
Ammonium		Ns	Ns	0.880P^∗^	ns	0.975P^∗^	ns	ns	ns
Total nitrogen					ns	0.977P^∗^	–0.938P^∗^	ns	ns
Growth rate						ns	ns	–0.982P^∗∗^	–0.983P^∗∗^
PME							–0.988P^∗^	0.968P^∗^	ns
Survival								–0.913P^∗^	ns
GSA									0.983P^∗∗^

*“ns” means there are no correlations between variables, ^∗^means variables significantly correlated at 0.05 level; ^∗∗^means variables significantly correlated at 0.01 level. PME, phosphomonoesterase; GSA, glutamine synthetase activity; NRA, nitrate reductase activity*.

## Discussion

The results showed that the growth and survival rates of *R. calicaris* var. *japonica* all decreased as nitrogen availability increased. Unlike lichens as already observed on other *Usnea* species (Carreras et al., 1998; Munzi et al., 2013), we didn’t find any fertilization effects on both survival and growth of *R. calicaris* var. *japonica*. The lichen growth rate under the 57.99 kg N ha^-1^.y^-1^ treatment was only 39.72% of that under the 8.59 kg N ha^-1^.y^-1^P treatment (taking the background nitrogen load into accounted), and this difference was significant (*p* < 0.05). The propagule survival rate was 76.35% under the 6.0 kg N ha^-1^.y^-1^ treatment, which was significantly lower than the 92.84% survival rate under the 0.0 kg N ha^-1^.y^-1^ (control). We suggested that this toxic effect was reasonable, and similar effect on non-vasculated epiphytes of South west part of China had been reported by Song et al. (2012). This effect can be attributed to a higher background of 16.7 kg ha^-1^.y^-1^ N load of China forest, which is about four times of the safe threshold of boreal forest system.

According to the survival response, we suggested that the N deposition threshold of *R. calicaris* var. *japonica* was 6.0 kg N ha^-1^.y^-1^. This was consistent with the results of thalli growth, because even the least nitrogen addition also led to a growth decrease when nitrogen load higher than control (8.59 kg N ha^-1^.y^-1^), which suggested that its threshold based on growth was lower than 8.59 kg N ha^-1^.y^-1^. Furthermore, in the ecology of high plants, seedlings usually are vulnerable than mature individual, then it was reasonable to infer 6.0 kg N ha^-1^y^-1^ as the threshold for *R. calicaris var. japonica*. In fact, to date, there are no reports on the use of lichen thalli growth and propagule mortality to determine N deposition threshold. We creatively introduced “survival” from population ecology of high plants into lichens, through which we were able to observe the difference among nitrogen treatments conveniently. Compared with our research, previous researches concerning lichen nitrogen sensitivity mostly exist on their physiological response (Munzi et al., 2009a,b, 2012), which cannot evaluate lichens’ N deposition threshold quantitively at all; in addition, N deposition thresholds of many ecosystems were derived from empirical methods and model simulation (Geiser and Neitlich, 2007; Fenn et al., 2008; Geiser et al., 2010), which are quite labor and time consuming. Our results showed that not only propagule survival rate of *R. calicaris* var. *japonica* was sensitive to nitrogen stress, but also demonstrated convenience in determination the concentration and deposition of N load, lichen biomass and survival, so our method might be reliable and applicable to future studies.

Our results indicated that *R. calicaris* var. *japonica* responded nitrogen stress in an integrated way. Previous research suggested that photosynthesis system (indicated by Fv/Fm, which is the maximum quantum efficiency of photosystem II, and OD_435_/OD_415_ of chlorophyll) and cell membrane of this lichen were damaged by nitrogen stress (Wang et al., 2015). Our results indicated that it had a strong capacity to adsorb ammonium N, which is consistent with many studies that lichens adsorb ammonium N more readily than nitrate N (Miller and Brown, 1999; Dahlman et al., 2004; Gaio-Oliveira et al., 2004; Toze et al., 2005). The results showed that *R. calicaris* var. *japonica* could adsorb ammonium N in NH_4_^+^ solution at a relatively lower concentration (0.04 mM), and the *V*_max_ was 0.43 mg.g^-1^. In contrast, NO_3_^-^N adsorption could not occur in *R. calicaris* var. *japonica* when the concentration of the NO_3_^-^ solution was lower than 0.4 mM and the corresponding *V*_max_ was only approximately 0.1 mg.g^-1^. Ammonium N adsorption in lichens is a passive process of ion exchange adsorption (Dahlman et al., 2004), then ammonium ions in solution can be rapidly bound to the extracellular space of the lichen thalli (Miller and Brown, 1999), so lichen species with more ion exchange sites would have stronger ammonium N adsorption capacity and thus be more susceptible to ammonium, which is thought to be one of the important mechanisms underlying N sensitivity in lichens (Gaio-Oliveira et al., 2005). In contrast, the adsorption of nitrate is an active process concerning oxygen and energy consumption (Crittenden, 1996), since nitrate, upon entering into cells, must be reduced to ammonium N before it can be utilized. Thus the cost of nitrate N utilization is rather higher than ammonium. Therefore, lichens tend to use ammonium N can be considered a consequence of long-term evolutionary adaptation. It should be noted that adsorption of both forms of N can occur in some lichen species (Johansson et al., 2010), so the ammonium N and nitrate N adsorption characteristics of lichens may be species specific.

The results showed that excessive application of N significantly damaged the balance between nitrogen and phosphorous of lichen thalli. We found an accumulation of NH_4_^+^-N, TN, TP but not that of NO_3_^-^-N. Specifically, NH_4_^+^-N and TN exhibited an overall increasing trend as N deposition increased. When the intensity of N deposition reached 57.99 kg N ha^-1^.y^-1^, the growth rate of the lichen was inhibited, and the content of ammonium N decreased. However, there were no significant differences in the nitrate N content among the treatments. Studies have shown that as N deposition increases, the N-sensitive lichen *Evernia prunastri* accumulates more ammonium N than the N-tolerant lichen *Xanthoria parietina*, while *X. parietina* accumulates more nitrate N than *E. prunastri* (Gaio-Oliveira et al., 2005). Thus, our results are consistent with that of N-sensitive lichens. The results also showed that when N deposition was lower than 32.99 kg N ha^-1^.y^-1^, the lichen P content exhibited an increasing trend, but when N deposition was greater than 50.0 kg N ha^-1^.y^-1^, the P content significantly decreased, which also resulted in a sharply increasing of N:P(>10.0). Our results agreed with the response of *Cladonia portentosa*, which showed that N deposition led to an increase in the N content and the N:P ratio (Hogan et al., 2010b). Furthermore, we found that activity of PME was elevated with an increasing nitrogen supply, which meant that P limitation occurred as it was well known that elevation of PME is a response of P deficiency. In addition, external P addition could double the growth of *Lobaria pulmonaria*, which was a species of cyanolichens could fix atmospheric nitrogen (McCune and Caldwell, 2009). Therefore, it was of high possibility that the *R. calicaris* var. *japonica* would experience a P deficiency under present nitrogen conditions.

Our results suggested that GSA elevated significantly with increased nitrogen availability, then decreased when nitrogen stress reached 1.6 mM. This GSA elevation was consistent to the results from high plants, which proved that external nitrogen supply can stimulate the expression of GS gene (Chen et al., 2010). The decrease of GSA implied a damage occurred when lichen were treated with higher nitrogen solution. In contrast, NRA was faintly influenced by external nitrogen supply, though it also could be stimulated by external nitrogen (Wang et al., 2003). The differentiated appearance of GSA and NRA could be owing to the difference in thalli adsorption and accumulation between ammonium and nitrate. This also demonstrated that lichen respond nitrogen stress in an integrated way.

## Conclusion

Nitrogen stress *did* impact growth and survival of *R. calicaris* var. *Japonica* in forest of Shennongjia Mountain, and its sensitive threshold indicated by the propagule survival rate was 6.0 kg N ha^-1^⋅y^-1^. *R. calicaris* var. japonica demonstrated an integrated physiological response to nitrogen stress, including selective adsorption between nitrate N and ammonium N, increased thalli N and P content, and P limitation, in addition with an activity elevation of PME and GSA.

## Author Contributions

C-HW designed the whole experiment, determined the study sites, analyzed the data, and wrote the manuscript. MW conducted the field experiment and participated in measuring the N and P content of lichen thalli. R-ZJ conducted the experiment of propagule survival. HG finished the assay of activity of PME, GS, and NR, and conducted the experiments of adsorption of ammonium and nitrate.

## Conflict of Interest Statement

The authors declare that the research was conducted in the absence of any commercial or financial relationships that could be construed as a potential conflict of interest.
